# Cheap and Nasty? The Potential Perils of Using Management Costs to Identify Global Conservation Priorities

**DOI:** 10.1371/journal.pone.0080893

**Published:** 2013-11-15

**Authors:** Erin McCreless, Piero Visconti, Josie Carwardine, Chris Wilcox, Robert J. Smith

**Affiliations:** 1 Department of Ecology and Evolutionary Biology, University of California Santa Cruz, Santa Cruz, California, United States of America; 2 Computational Ecology and Environmental Science Group, Microsoft Research, Cambridge, United Kingdom; 3 Global Mammal Assessment Program, Department of Biology and Biotechnologies, Sapienza Università di, Roma, Rome, Italy; 4 CSIRO Sustainable Ecosystems, Dutton Park, Queensland, Australia; 5 CSIRO Marine and Atmospheric Research, Hobart, Tasmania, Australia; 6 Durrell Institute of Conservation and Ecology, University of Kent, Canterbury, Kent, United Kingdom; Bangor University, United Kingdom

## Abstract

The financial cost of biodiversity conservation varies widely around the world and such costs should be considered when identifying countries to best focus conservation investments. Previous global prioritizations have been based on global models for protected area management costs, but this metric may be related to other factors that negatively influence the effectiveness and social impacts of conservation. Here we investigate such relationships and first show that countries with low predicted costs are less politically stable. Local support and capacity can mitigate the impacts of such instability, but we also found that these countries have less civil society involvement in conservation. Therefore, externally funded projects in these countries must rely on government agencies for implementation. This can be problematic, as our analyses show that governments in countries with low predicted costs score poorly on indices of corruption, bureaucratic quality and human rights. Taken together, our results demonstrate that using national-level estimates for protected area management costs to set global conservation priorities is simplistic, as projects in apparently low-cost countries are less likely to succeed and more likely to have negative impacts on people. We identify the need for an improved approach to develop global conservation cost metrics that better capture the true costs of avoiding or overcoming such problems. Critically, conservation scientists must engage with practitioners to better understand and implement context-specific solutions. This approach assumes that measures of conservation costs, like measures of conservation value, are organization specific, and would bring a much-needed focus on reducing the negative impacts of conservation to develop projects that benefit people and biodiversity.

## Introduction

Biodiversity is declining at a rapid rate [1] but there is little spatial overlap at a global level between conservation need and local funding availability. Many countries therefore rely on funds from international donors to conserve biodiversity [2], and a number of studies have developed prioritization schemes to identify the most cost-effective places for doing so [3]. The first and best known of these schemes were developed by international conservation non-governmental organizations (NGOs) and based on patterns of biodiversity distribution and threat [4–6] to identify and fundraise for priority regions [7]. However, these systems attracted criticism for their theoretical weaknesses and the coarse spatial resolution of the underlying biodiversity data [8,9]. Subsequent schemes used more robust methods and fine-resolution biodiversity data to identify finer-scale priority areas [10–12]. Finally, global conservation prioritization became more systematic with the emergence of target- and complementarity-based approaches [13–16].

These early analyses were instrumental in shifting the focus from single species to more holistic biodiversity conservation schemes [17], but fell short of identifying cost-efficient priorities because they did not account for spatial variation in conservation costs [9]. Similar analyses at the sub-national scale moved to overcome this issue by including acquisition, management or opportunity cost data [18,19]. These costs could then be combined with biodiversity values to calculate and compare the expected return on investment for a range of potential conservation actions [20,21]. Return-on-investment approaches were subsequently applied at a global scale, using models that predicted protected area management costs by country based on each country’s purchasing power parity (PPP) and gross national income (GNI) [22,23]. This work revealed that variation in protected area management costs overwhelmed the effects of biodiversity and threat in determining global conservation priorities, largely because country-level costs ranged by seven orders of magnitude [24,25]. 

The strong influence of management cost on global prioritizations highlights the importance of using a cost metric that reflects the true, realized costs of achieving conservation goals [26,27]. However, to our knowledge there has not yet been a broad-scale assessment of how well the most widely used global cost estimates of protected area management predict the realized costs of project implementation. Ideally, global conservation cost metrics should reflect the probability of long-term project success because: a) obstacles to implementation decrease the likelihood of attaining conservation objectives, and b) overcoming these obstacles will increase project costs. Many countries with low predicted costs are developing countries [28] where less stable socio-political environments might make conservation investments risky. Foreign investors often avoid these countries due to their unreliable business environments [29–31], and the same factors that deter economic investments likely present challenges to conservation as well.

Successful project implementation depends on a range of factors beyond the direct costs of staff wages, equipment and time [26,32]. For example, civil society involvement and support are critical to conservation success [7,33]. When international agencies work in foreign countries but fail to engage local communities, they must rely on national governments to manage funds and implement projects. This reliance on governments can be problematic in countries where political institutions are weak, unstable, or corrupt, as projects will be vulnerable to changes in or failures of national governments or economies [34,35]. Without strong local capacity and support, outside organizations may not be able to carry projects forward in times of instability, or may face ongoing resistance from local communities whose interests are not well represented. Thus, failure to engage civil society may reduce the long-term success of conservation initiatives. These issues can be mitigated or avoided by working closely with local stakeholders through all stages of planning and implementation [7], but the extent to which these efforts increase project costs is difficult to predict and has not been well studied. 

Conservation planning efforts must also consider implications for local people, as any negative impacts can reduce human wellbeing and erode support for conservation. This is particularly important because many nations with low predicted management costs are developing countries with large populations of rural, disenfranchised poor [36,37]. It is often these people who bear the brunt of conservation opportunity costs [38–40] through the loss of agricultural land, limitations on infrastructure development and restrictions on harvesting wild species [41]. Indeed, strict enforcement of protected area policies can result in the displacement or eviction of these people from their land [42–44], sometimes involving the illegal use of force [45].

Conservation NGOs are committed to improving project success and minimizing negative impacts on project stakeholders, but the costs of achieving these goals are rarely considered in global prioritization exercises. There is, therefore, a need to investigate whether using modeled management costs in global conservation prioritizations highlights countries where implementation is more difficult and negative impacts on people more likely. Here, we investigate the relationships between national-level protected area management costs and a range of factors that broadly relate to conservation effectiveness and impacts on people.

Conservation actions involve a range of costs beyond direct management costs, including acquisition, transaction, damage, and opportunity costs [19]. In this analysis we focus only on management costs for several reasons. Our broad goal here is to investigate issues relating to where international conservation donors should spend money to have the greatest, most cost-effective impact on conservation. Management cost is the metric most often used in global-scale conservation prioritization [22–24,26,46], and is an ongoing cost that is directly related to the success or failure of conservation interventions. Using acquisition cost is inappropriate in many places where land cannot be bought or sold [47], and to our knowledge transaction and damage costs have not been studied or modeled at a global scale. Global data on opportunity costs do exist [48], and like management costs these costs are ongoing, but estimates of opportunity costs are generally based on agricultural value and are only weakly related to conservation effectiveness. In addition, many international donors are interested in funding existing protected areas, where measures of agricultural opportunity costs are not relevant. Moreover, because acquisition, transaction, and damage costs are one-off costs, they will be outweighed in the long term by the ongoing costs of management. Therefore, management costs are the most important cost type for international donors to consider in the initial stages of global prioritization.

In this paper we compare modeled country-level conservation management costs to widely used global indicators for civil society involvement in conservation, governance, and human rights. We hypothesize that countries with low predicted management costs also tend to have lower levels of civil society involvement in conservation, less effective and stable governments, and less protection of human rights. Support of these hypotheses would suggest that the management cost estimates most often used in global conservation prioritization are simplistic and neglect important factors that impact project implementation and outcomes. Therefore, achieving long-term conservation success in countries with low predicted costs, and avoiding unintended negative impacts on people, may be more difficult or more expensive than current cost models predict.

## Methods

### Conservation management cost data

Our analysis adopted the most widely used approach for calculating the protected area management costs for each country, the Balmford-Moore equation [22,23]. This model has been criticized for inflating the predicted management costs of conservation in the more expensive countries, as the original data for developed countries included habitat restoration and other more intensive management actions [49]. However, it remains the primary approach for estimating national-level management costs and is the basis of most global cost-based conservation prioritization schemes published in the literature [22–24,46]. 

The Balmford-Moore equation states that the cost of managing land as protected areas is a non-linear function of the nation’s gross national income (GNI) scaled by its total area, its purchasing power parity (PPP), and the size of a protected area (PA):

$$\begin{array}{c} log_{10}\left(annual\;cost,\;US\$\;km^{-1}\right)\;=\;\\ 1.765\;-\;0.299*log_{10}\left(PA,\;km^{2}\right)\;+\;1.014*log_{10}\left(PPP\right)\;+\;\\ 0.531*log_{10}\left(GNI,\;US\$\;km^{-2}\right)\;-\;0.771*log_{10}\left(PA,\;km^{2}\right)*log_{10}\left(PPP\right) \end{array}$$

For this analysis we used a standard reserve size of 100 km^2^ for all countries to enable comparison of conservation costs for equal-sized protected areas across countries. While data on the extent of protected areas exist for most countries [50], we used a standardized area for all countries because this method is better suited to our goal of comparing the standard cost metrics used in global prioritizations against several other national-level socio-political indicators. Data for GNI, PPP, and country land area were obtained from the United Nations Statistics Division (unstats.un.org) and are available in the File S1.

### Civil society involvement in conservation data

The extent to which civil society is involved in conservation efforts is difficult to measure and quantify, particularly at the national level. To our knowledge, there are three datasets that provide unbiased, quantifiable, and comparable information about this issue for many countries globally (i-iii below). We used all of these datasets in an effort to capture as much information as possible regarding civil society involvement in conservation. All data are available in Files S2, S3, and S4.

#### i): Membership in BirdLife International partner organizations

BirdLife International (BLI) is the world’s largest global partnership of conservation organizations, maintaining partnerships with local, independent, membership-based NGOs in over 100 countries on all inhabited continents. Data on citizen membership for BirdLife partner NGOs is available online for 86 of these countries (www.birdlife.org/worldwide/national/index.html; File S2). While BLI is a UK-based NGO, we detected no bias toward the existence of more BLI partnerships in former British colonies, and countries with BLI partners are widely distributed across all continents. To compare membership between countries, we standardized the data by dividing NGO membership by the country’s total population (population data obtained from unstats.un.org; File S3). The resulting value is the proportion of a country’s population that belongs to a leading local conservation NGO.

#### ii): IUCN member organizations

The International Union for the Conservation of Nature (IUCN) is the world’s oldest and largest global environmental organization, with more than 1200 member organizations (200+ government and 900+ non-government) in 160 countries (www.iucn.org). The number of IUCN organizations per million people was one of many variables used to create the Environmental Sustainability Index (ESI) [51]. We obtained values directly from the ESI (www.yale.edu/esi/c_variableprofiles.pdf; File S4).

#### iii): Local Agenda 21 Initiatives

Agenda 21 is a voluntary action plan of the UN regarding sustainable development, intended to motivate action at international, national, regional, and local levels (http://www.un.org/esa/dsd/agenda21/). Local Agenda 21 initiatives are measures undertaken and overseen by local authorities to address problems of environmental sustainability, and represent the involvement of civil society in environmental governance. The number of Local Agenda 21 initiatives per million people was another variable calculated for the 2005 ESI [51], and we used these values in our analyses (www.yale.edu/esi/c_variableprofiles.pdf; File S4).

### Governance data

One of the most widely used systems for measuring quality of governance at the national level is the Worldwide Governance Indicators (WGI), constructed by the World Bank and based on consultations and surveys with citizens, experts, businesses, and international organizations [52]. We used three of the six “dimensions of governance” assessed by the WGI: Political Stability and Absence of Violence, Control of Corruption, and Government Effectiveness. All data and background information for each index are available online (info.worldbank.org/governance/wgi/sc_country.asp) and in File S5.

### Human rights data

The most comprehensive, national-level global dataset on protection of human rights is the Cingranelli-Richards Human Rights Dataset (CIRI) [53]. The CIRI dataset contains standards-based, quantitative information on government respect for a wide range of human rights in most countries. In our analyses we used the Empowerment Rights Index, an additive index constructed from seven individual indicators: Foreign Movement, Domestic Movement, Freedom of Speech, Freedom of Assembly and Association, Workers’ Rights, Electoral Self-Determination, and Freedom of Religion. The index ranges from 0 (no government respect for these seven rights) to 14 (full government respect for these seven rights). Data are available online (www.humanrightsdata.org) and in File S6.

For all datasets we used the most recent data available. Years of the data range from 2001 (Local Agenda 21 initiatives) to 2012 (membership in BLI partner organizations). 

### Statistical analysis

We separately examined the relationships between national-level modeled protected area management costs and each socio-political variable: civil society involvement in conservation (NGO membership, IUCN Organizations, and Agenda 21 Initiatives), quality of governance (bureaucratic effectiveness, control of corruption, and political stability), and human rights (Empowerment Rights Index). We also investigated the relationships between each of the three measures of civil society involvement. Because none of the individual datasets satisfy the normality assumptions for parametric correlation, we used nonparametric Spearman rank correlations for all analyses. 

Analyses were performed in the statistical packages JMP [54] and R [55].

## Results

**Figure 1:**
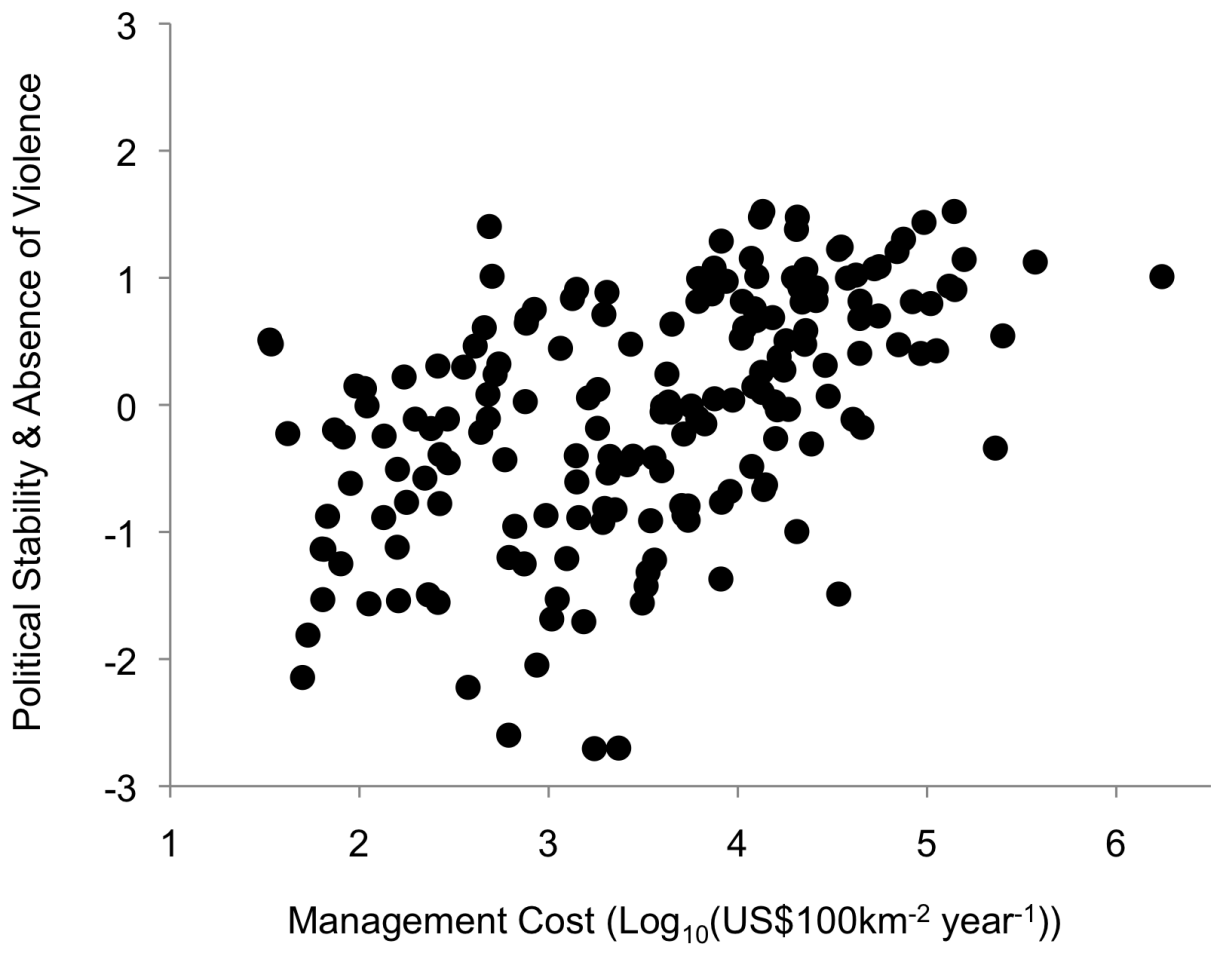
Plot of predicted conservation management cost (log_10_ transformed) vs. index for Political Stability and Absence of Violence (*N*=184).

The modeled costs of managing a 100km^2^ terrestrial protected area in each country ranges between US$34 for Mongolia and US$1.7 million for Monaco, with a median value of US$4,260. Political stability is correlated with protected area management costs (*N*=184, Spearman ρ=0.5071, p<0.0001; Figure 1): estimates for management costs in the ten most stable countries are 82 times higher than in the ten least stable. 

**Figure 2:**
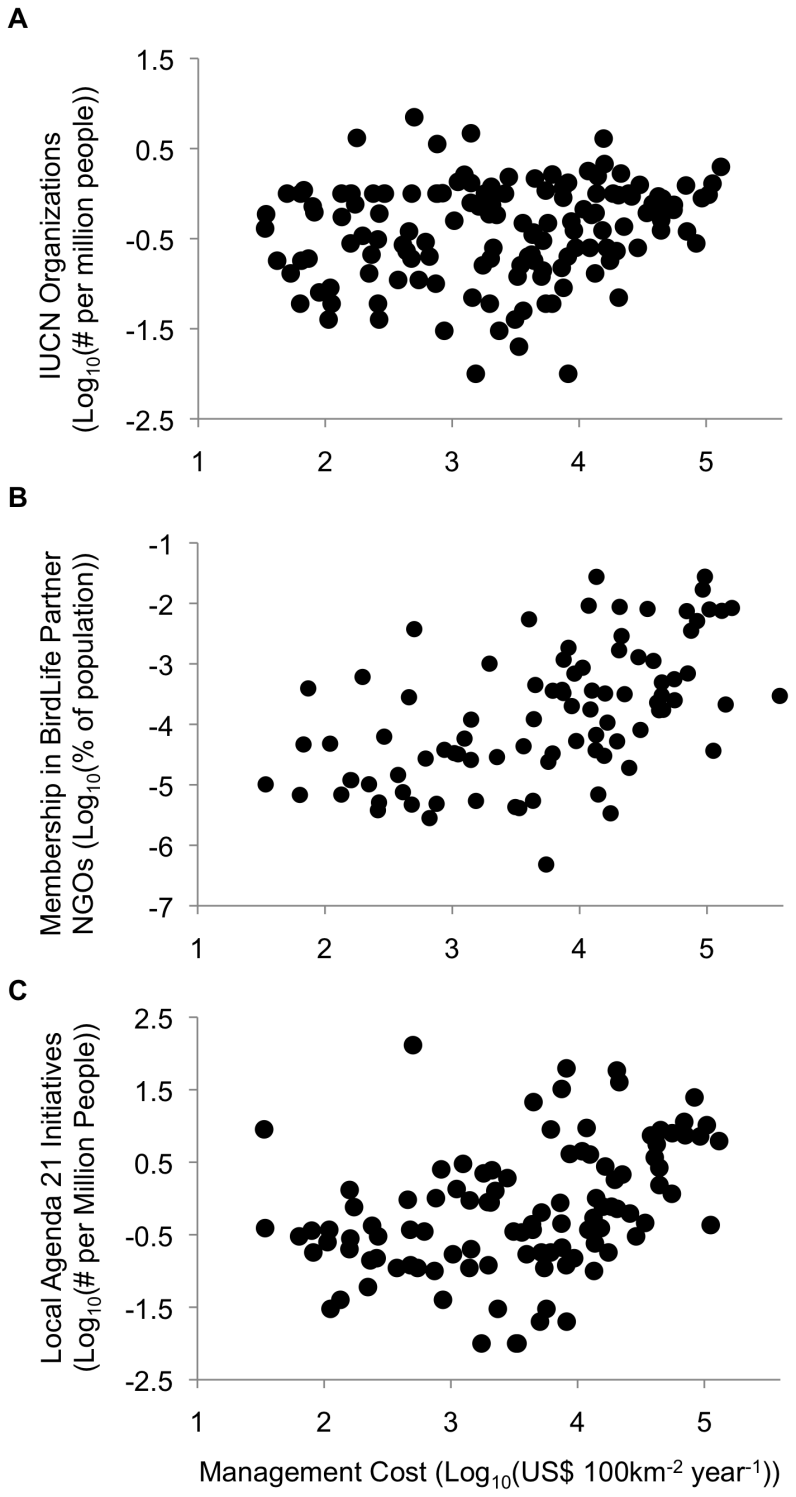
Plots of predicted conservation management cost vs. three metrics of civil society engagement in conservation action (all values log_10_ transformed): (*a*) Proportion of a country’s population belonging to BirdLife International partner NGO (*N*=89); (*b*) IUCN member organizations per million people (*N*=140); (*c*) Local Agenda 21 initiatives per million people (*N*=104).

We found positive correlations between each of the metrics for civil society involvement in conservation (Table 1), and between conservation management cost and each of these metrics (Table 2, Figure 2). Together, these analyses suggest that populations are less involved in conservation efforts in countries with low modeled management costs. 

**Table 1:** Results of Spearman rank correlation analyses comparing each metric for civil society involvement in conservation.

**Variable comparison**	***N***	**Spearman ρ**	**p-value**
NGO Membership & IUCN Organizations	80	0.5524	<0.0001
IUCN Organizations & Agenda 21 Initiatives	106	0.6338	<0.0001
NGO Membership & Agenda 21 Initiatives	62	0.7666	<0.0001

**Table 2:** Results of Spearman rank correlation analyses comparing predicted conservation cost (US$ 100km^-2^ year^-1^) with each metric for civil society involvement in conservation.

**Civil society involvement metric**	***N***	**Spearman ρ**	***p*-value**
NGO Membership	89	0.5728	<0.0001
IUCN Organizations	140	0.3369	<0.0001
Agenda 21 Initiatives	104	0.4474	<0.0001

**Figure 3:**
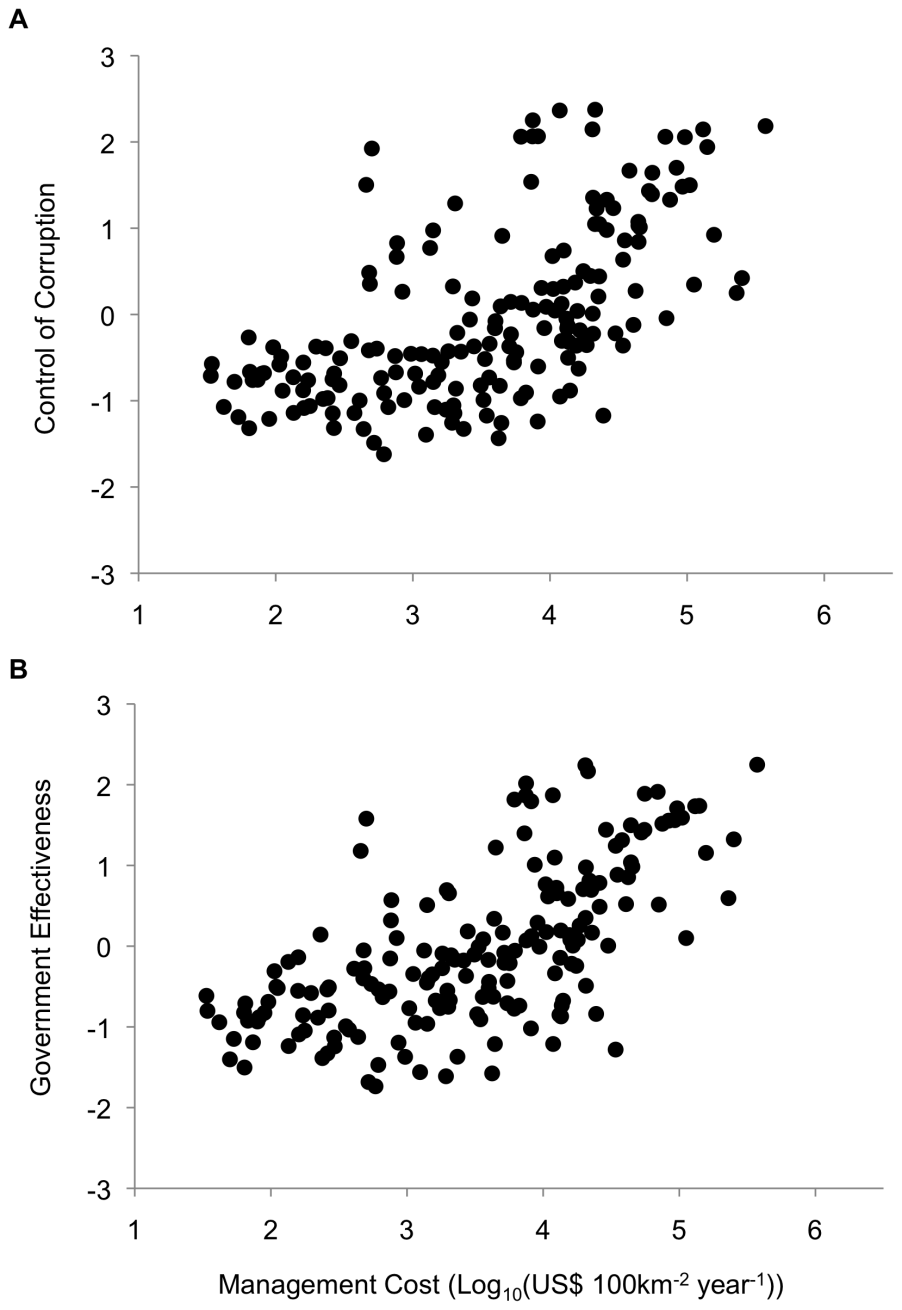
Plots of predicted conservation management cost (log_10_ transformed) and indices for: (*a*) Control of Corruption (*N*=184); (*b*) Government Effectiveness (*N*=184).

We found positive correlations between predicted conservation cost and control of corruption (*N*=184, Spearman ρ=0.6401, *p*<0.0001; Figure 3a): protected area management costs in the ten least corrupt countries are 41 times higher than in the 10 most corrupt countries. Governments are also less effective in low-cost countries (*N*=184, Spearman ρ=0.6729, *p*<0.0001; Figure 3b): protected area management costs in the ten countries with the most effective governments are 56 times higher than in the ten with the least effective governments.

**Figure 4:**
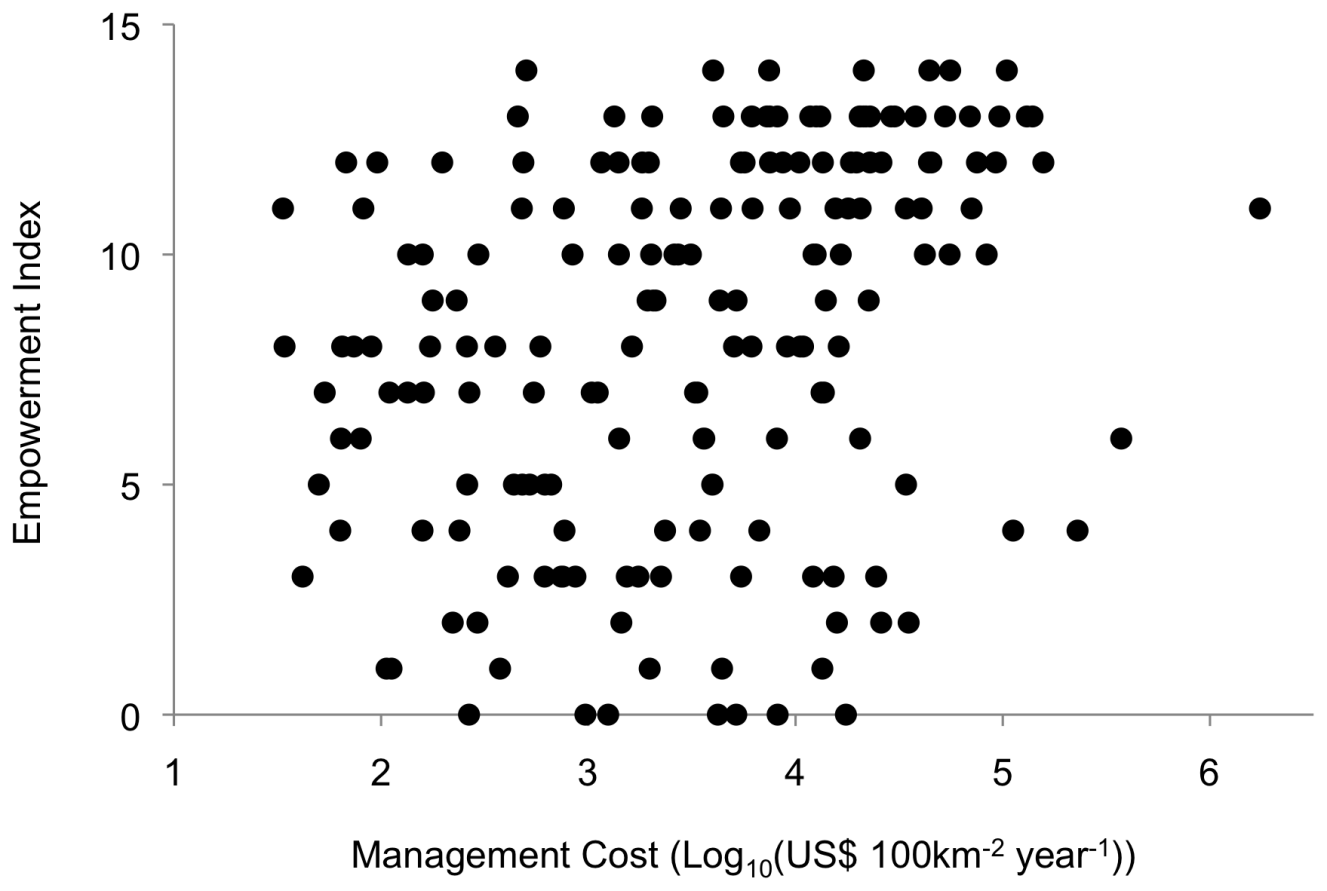
Plot of predicted conservation management cost (log_10_ transformed) and Empowerment Rights Index (*N*=181).

There is a positive correlation between conservation cost and protection of human rights (*N*=181, Spearman ρ=0.3720, *p*<0.0001; Figure 4): countries with low protected area management costs generally have poorer human rights records, so that the seven countries sharing the highest Empowerment Rights Index score are 6.3 times more expensive than the seven countries sharing the lowest score.

## Discussion

This analysis investigates the broader implications of a commonly proposed approach for identifying ‘cost-efficient’ globally important areas for conservation investment. Our analysis shows that national-level estimates for conservation management cost are correlated with socio-economic and governance issues that can affect conservation costs and outcomes. Our results suggest that conservation investments in countries with low predicted management costs could be prone to a suite of negative outcomes that increase the realized cost of conservation actions and make some projects less likely to succeed. 

Our first result (Figure 1) shows that political instability is higher in countries with low modeled conservation management costs. This instability is a recognized problem in conservation, where it is linked with breakdowns in local or national institutional support for projects, threats to project management and enforcement staff, and biodiversity loss through the impacts of militias and refugees [56]. It is also a prevalent problem, as 81% of violent conflicts between 1950 and 2000 took place completely or partially within biodiversity hotspots [57]. 

Case studies have shown the impacts of political instability on conservation can be mitigated by support from the local population [34,58], but our results also showed that civil society involvement is lower in these less stable countries (Figure 2; Table 2). Local capacity and involvement in conservation efforts is a complex social phenomenon that varies widely within countries as well as between them, and the three metrics we used to quantify this variable are only broad-scale approximations of local involvement at the country level. However, to our knowledge these are the only multi-country datasets that provide useful and quantifiable measures for this issue. Each index addresses a slightly different aspect of local involvement, and together they represent the conservation-related activities of local people and governments through both international NGOs (BLI partners) and multilateral agencies (IUCN organizations and UN Agenda 21 Initiatives). The significant correlations we found between the metrics (Table 1) indicate that they capture complementary features of local involvement that tend to vary together across countries globally. Our study does not address the reasons that people are less involved in some countries, or the consequences of this lower level of involvement. However, both anecdotal evidence and formal studies demonstrate that conservation efforts are more successful when local people are engaged in the process [59]. Thus, if conservation interventions are to succeed in many countries with lower management costs, external donors may need to spend more time and money developing relationships at the local level and building and maintaining project support.

This lower involvement of civil society in countries with low predicted management costs highlights another problem with using these cost estimates as a metric for realized conservation costs: volunteering and local fundraising are likely to be higher in countries with an engaged civil society [3]. Even more importantly, externally funded projects in low-cost countries must rely heavily on local and national government agencies for implementation. This is worrying in light of our results (Figure 3a), which indicate that governments in low-cost nations are more corrupt, and the results of earlier studies showing that conservation success is lower in corrupt countries [35,60]. The issue might not affect estimates of direct working costs, as recent research suggests the relatively weak purchasing power in corrupt countries still makes conservation projects cost effective [61]. However, accounting for the quality of governance in addition to biodiversity and direct costs can change cost-effective investment priorities at a global scale [62], and a number of case studies have shown the impacts of corruption often involve more than an increase in direct costs. For example, the misappropriation of conservation benefits or exposure to rent-seeking officials often results in loss of local support and project failure [63,64].

We found that countries with low predicted costs score poorly on bureaucratic effectiveness measures (Figure 3b). This is a critical result, given that a wide range of conservation initiatives rely on effective bureaucratic systems for implementation: most protected areas are state-managed, many donor-funded projects work with and through local or national governments, and community based conservation relies on government support. The impacts of this problem likely extend beyond short-term project effectiveness, as the success of conservation initiatives often depends on changing institutional, economic and socio-ecological frameworks [34]. Indeed, many of conservation’s negative impacts on people, from poorly implemented compensation or resettlement programs to the capture of ecotourism resources by local elites, are related to this issue [65–69]. Moreover, the literature shows that such negative interactions with the state are not always indirect, as there are a number of documented human rights abuses that arose from heavy-handed enforcement of conservation policy [70]. This is why our final result (Figure 4) – that low-cost countries also have the worst human rights records – is particularly troubling. The implications of this finding are obvious: relying on governments with poor human rights records to achieve action on the ground is more likely to negatively impact people.

Taken together, our results suggest that using country-level estimates of protected area management costs in global conservation prioritization, without accounting for other related factors, is problematic. Projects in apparently low-cost countries could be less likely to succeed, more expensive to implement than originally expected, and more likely to have negative impacts on local people. Some donors already implicitly recognize the importance of accounting for implementation factors when directing funds, by avoiding difficult countries or working where they have historically built long-term collaborations and already have local support [71]. This may partly explain why the published fine-scale global prioritization schemes have had a relatively minor influence on conservation policy [72,73]. There is a real need, therefore, to move beyond the simplistic assumption that there can be one globally relevant equation for estimating the long-term costs of successful conservation implementation and management over entire countries, and to develop more nuanced and context-specific cost metrics. 

Efforts to include broader socio-political factors in cost estimates must be undertaken with caution for two major reasons. First, recent studies at sub-national scales that accounted for some cost types and implementation factors, such as opportunity costs and local support, reveal complex and dynamic relationships between costs, biodiversity values and favorable conditions for conservation [40,47]. The non-biological factors that influence conservation opportunities and outcomes, and the relative importance of these factors over different temporal and spatial scales, are highly variable but are known to be essential for achieving both biodiversity conservation and human wellbeing [74–76]. Developing reliable and broadly applicable metrics for realized costs, and understanding the broader factors that influence conservation opportunity and outcomes but are not captured in cost estimates, will require an intensive research effort to better understand the nature of these relationships both within and between countries.

Second, caution must be used when generating global cost metrics that include data on potential negative impacts on people. Traditionally, developing such global metrics has involved converting different factors into a common currency, such as predicting lost revenues to local people by calculating opportunity costs [40], or considering these negative impacts independently and then investigating trade-offs or returns on investment [77]. This is a reasonable method when accounting for factors such as local fundraising, volunteering and the direct impacts of political instability, corruption and bureaucratic quality [26,32,62]. However, measures of human welfare or human rights cannot be considered simply as factors to be traded off against biodiversity benefits. Instead, there is a need to gather data to better understand the economic costs of avoiding negative impacts. Current prioritization approaches fail to capture the strategies used by conservation organizations to reduce the risk of negatively impacting people, such as changing their approach in high-risk countries or building long-term relationships with existing local partners. Many organizations do this in an *ad hoc* manner, and would benefit from adopting a more systematic approach. Producing a relevant cost metric for such systematic analyses will involve capturing types of information that are rarely used in conservation planning. The critical next step is for conservation scientists to engage with practitioners in order to produce more robust prioritization schemes that are based on explicitly defined goals and appropriate measures of conservation costs, opportunities and threats [78,79].

## Conclusions

We have long recognized there is no one global measure of conservation value, as different organizations favor different aspects of biodiversity [4]. However, there is an implicit assumption in the literature that there can be a single global measure of conservation costs and that estimates of direct conservation cost based on macro-economic differences automatically make working in some countries better value for money. Our work reveals this assumption to be simplistic, because these basic economic indicators are linked with factors that make implementation more difficult and negative impacts on people more likely. Overcoming these challenges will add to the cost of implementation in predicted low-cost countries. Conservation donors and organizations are well aware of the difficulties and tailor their projects accordingly [79], but this makes any estimate of their projects’ costs just as organization-specific as their measures of conservation value. Calculating the total, realized cost of conservation efforts remains vital for decision making, and the need to produce these data adds to the list of compelling reasons for conservation researchers to collaborate closely with practitioners [7]. 

## Supporting Information

File S1 **Country area (2010), gross national income (GNI; 2007-2010), purchasing power parity (PPP; 2005-2009), and calculated values for Balmford-Moore conservation management cost by country.** Area, GNI, and PPP data are from the United Nations Statistics Division (unstats.un.org).
(CSV)

File S2 **Citizen membership in BirdLife International (BLI) partner NGOs by country (2012).**Data are from BLI (www.birdlife.org/worldwide/national/index.html).
(CSV)

File S3 **Country population data (2010).**Data are from United Nations Statistics Division (unstats.un.org).
(CSV)

File S4 **Environmental Sustainability Index (ESI) values for IUCN Organizations per million people (2003-2004) and Local Agenda 21 Initiatives per million people (2001) by country.**Data are from ESI 2005 (www.yale.edu/esi/c_variableprofiles.pdf).
(CSV)

File S5 **World Governance Indicators for Control of Corruption, Government Effectiveness, and Political Stability & Absence of Violence by country (2010).**Data are from the World Bank (info.worldbank.org/governance/wgi/index.asp).
(CSV)

File S6 **Cingranelli-Richards (CIRI) human rights data for Empowerment Rights Index by country (2010).**Data are from CIRI (www.humanrightsdata.org/).
(CSV)
